# TTC3-Mediated Protein Quality Control, A Potential Mechanism for Cognitive Impairment

**DOI:** 10.1007/s10571-021-01060-z

**Published:** 2021-02-27

**Authors:** Xu Zhou, Xiongjin Chen, Tingting Hong, Miaoping Zhang, Yujie Cai, Lili Cui

**Affiliations:** Guangdong Key Laboratory of Age-Related Cardiac and Cerebral Diseases, Affiliated Hospital of Guangdong Medical University, No.57, Renmindadaonan Road, Xiashan District, Zhanjiang, China

**Keywords:** TTC3, Protein quality control, Cognitive impairment, Down’s syndrome, E3 ubiquitin ligase

## Abstract

The tetrapeptide repeat domain 3 (*TTC3*) gene falls within Down's syndrome (DS) critical region. Cognitive impairment is a common phenotype of DS and Alzheimer’s disease (AD), and overexpression of *TTC3* can accelerate cognitive decline, but the specific mechanism is unknown. The TTC3-mediated protein quality control (PQC) mechanism, similar to the PQC system, is divided into three parts: it acts as a cochaperone to assist proteins in folding correctly; it acts as an E3 ubiquitin ligase (E3s) involved in protein degradation processes through the ubiquitin–proteasome system (UPS); and it may also eventually cause autophagy by affecting mitochondrial function. Thus, this article reviews the research progress on the structure, function, and metabolism of TTC3, including the recent research progress on TTC3 in DS and AD; the role of TTC3 in cognitive impairment through PQC in combination with the abovementioned attributes of TTC3; and the potential targets of TTC3 in the treatment of such diseases.

## Introduction

Down’s syndrome (DS) is also known as trisomy 21 syndrome because complete or partial trisomy of human chromosome 21 (Hsa21) is the main pathogenic event involved in DS. Current methods used to study trisomy 21, include detection of the Down’s syndrome critical region (DSCR), which contains many genes such as the tetrapeptide repeat domain 3 (*TTC3*) gene, that can lead to phenotypic features of DS (Kong et al. 2015; Sun et al. 2014). The DSCR concept was proposed in 1992 (Delabar et al. 1993) and refers to a region within 21q22.1-21q22.3 on Hsa21 (Park and Chung 2013). Researchers have identified 25 areas in this region associated with the DS phenotype, most of which are concentrated in the ~ 85 Kb range, and it has been proposed that more than one DSCR may exist (Dahmane et al. 1998; Sinet et al. 1994). These areas are also known as "sensitive areas" (Lyle et al. 2009). In addition, researchers believe that DSCR can be defined as a region on chromosome 21q22.12-q22.2 that contains DSCR1 (RCAN1), DYRK1A, DSCAM, and APP (Arron et al. 2006; Ronan et al. 2007). Some researchers used the GeneEntrez database to obtain 19 candidate genes for the Down syndrome critical region (DSCR) and studied the transcript levels and expression of these genes in the brain (Montoya et al. 2014). However, it is still unclear how many candidate genes for DSCR are exact.

*TTC3*, also known as *TPRDIII*, *RNF105*, and *DCRR1*, was first discovered in 1996 (Ohira et al. 1996; Tsukahara et al. 1996). Subsequently, the sequencing of chromosome 21 was completed in 2000, and the *TPRD* gene was renamed *TTC3* (Hattori et al. 2000). *TTC3* is located at 21q22.2 (Eki et al. 1997; Tsukahara et al. 1996). Some studies have investigated the effect of *TTC3* on neuron-related phenotypes, mostly in DS and Alzheimer’s disease (AD). As early as 1996, Tsukahara et al. (Tsukahara et al. 1996) proposed that *TPRD* (renamed *TTC3*) overexpression may contribute to morphological abnormalities in patients with DS. AD is closely related to DS (Startin et al. 2019; Wiseman et al. 2015; Zis and Strydom 2018). Most individuals with DS develop AD-related neuropathological changes after middle age, including the deposition of senile plaques, neurofibrillary tangles and abnormal accumulation of proteins (Mann 1988). Mutation of the *TTC3* gene may have a fundamental effect on an individual's cognitive function and learning ability (Kohli et al. 2016; Smith et al. 1997; Suizu et al. 2009), and the nerve damage caused by *TTC3* could be reversible (Berto et al. 2007, 2014).

In addition, the TTC3 protein encoded by *TTC3* is an E3 ubiquitin ligase (E3s) that contains a RING finger domain (RING E3s) and may affect protein degradation through ubiquitination (Gong et al. 2017). The TTC3 protein can promote the formation of protein aggregates when overexpressed and can affect the ubiquitination level and degradation rate of proteins via E3s activity, which may contribute to the aggregation of pathological proteins (Kim et al. 2019; Kong et al. 2020). We know that polypeptide chains are prone to misfolding from the beginning of synthesis, while E3s can maintain protein quality control (PQC) by selective ubiquitination and targeting misfolded proteins for degradation, thereby preventing protein aggregation and associated pathological phenotypes (Mishra et al. 2019; Samant et al. 2018). PQC can help organisms cope with the excessive aggregation of proteins (Costa-Mattioli and Walter 2020). The PQC system is an efficient system composed mainly of three parts: chaperones, the ubiquitin–proteasome system (UPS), and autophagy (Ciechanover and Kwon 2017; Joshi et al. 2016). Among them, chaperones are responsible for rescuing misfolded proteins (Freilich et al. 2018; Hipp et al. 2019); however, when various homeostasis in the cell is dysregulated and the efficiency of chaperones is affected, the UPS and autophagy remove a large number of aggregates that are not degraded by chaperones (Dikic 2017; Harris et al. 2020; Kabir et al. 2020), and in this process, E3s are key controllers of these shunts (Enam et al. 2018). Interestingly, TTC3 has E3s activity, and it has been reported to act as a cochaperone that can link chaperones and the UPS (Imai et al. 2003) and affect mitochondrial function; therefore, TTC3 has the potential to trigger autophagy (Gong et al. 2017; Kong et al. 2020). Taken together, the evidence is compelling that TTC3 has some overlap with PQC.

**Fig. 1:**
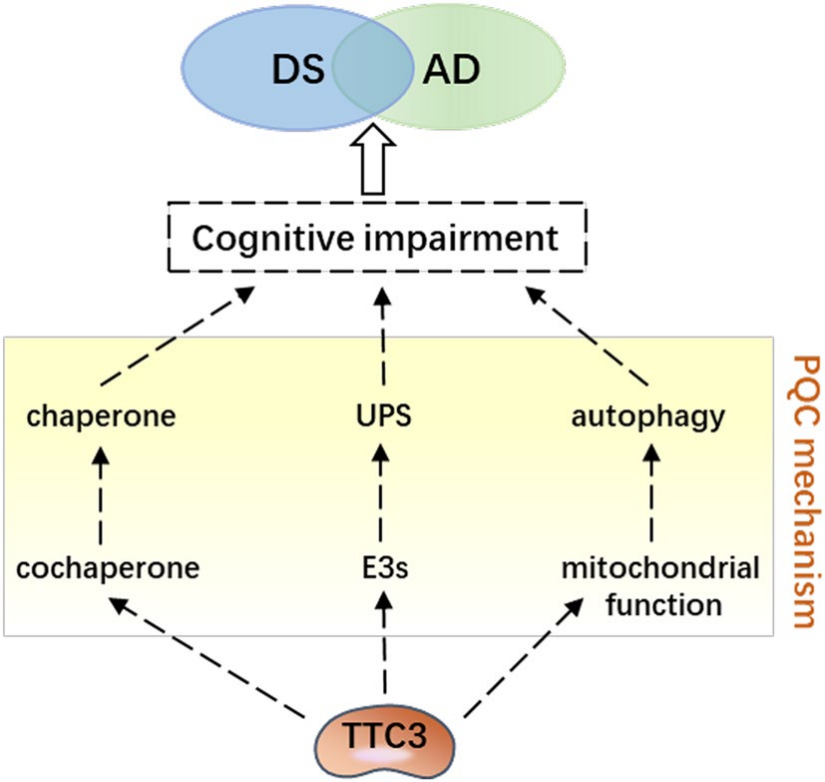
TTC3 and cognitive impairment The involvement of TTC3 in cognitive impairment may rely on PQC mechanisms. TTC3 functions as both a molecular cochaperone, ubiquitin E3 ligase, and major regulator of mitochondrial function

Since the accumulation of misfolded proteins promotes protein aggregation and neuronal death (Krstic and Knuesel 2013; Martinez-Cue and Rueda 2020; Uddin et al. 2020), the PQC system, which can counteract misfolded protein accumulation, is thought to be closely related to neuronal survival (Cristofani et al. 2017). An increasing number of studies show that E3s (Lu et al. 2017; Wei et al. 2016) and PQC (Tiernan et al. 2016; Verheijen et al. 2018) both play important roles in the pathogenesis of cognitive impairment; however, how E3 participates through PQC has not been reported. Although TTC3 has been reported to impair cognitive function (Montoya et al. 2014) and can affect protein degradation processes, it is unclear how TTC3 affects cognition through PQC. In this review, we collate and summarize the above evidence on TTC3 and its regulation of the protein degradation processes in cognitive impairment; then, we try to elucidate the important role of TTC3 in PQC and its potential as a therapeutic target approach to reduce cognitive decline (Fig. 1).

## Structural and Functional Features of TTC3

**Fig. 2:**
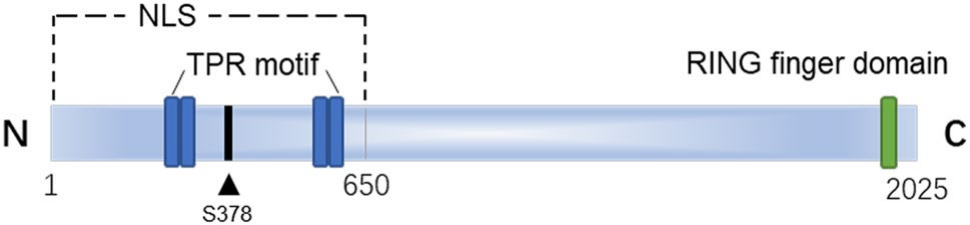
Structure and composition of TTC3 Four TPR motifs of TTC3 are located at 231–264 aa, 266–298 aa, 536–572 aa, and 576–609 aa. The RING finger domain is located at 1957–1997 aa. Ser378 is a possible Akt phosphorylation site. NLS is only known to be located in the 1–650 aa range

The TTC3 protein is 2025 amino acids (aa) in length and approximately 23 kDa in size, and it harbors two pairs of TPR motifs at the N-terminus and a RING finger domain at the C-terminus (Lamb et al. 1995; Ohira et al. 1996). In addition, it harbors a possible Akt phosphorylation site and an NLS site (Suizu et al. 2009) (Fig. 2). Phosphorylation sites and the NLS are auxiliaries that aid TTC3 in carrying out its physiological function. TPR motifs and RING finger domains are the primary functional domains, and they complement each other to complete the main physiological functions of TTC3. On the one hand, these domains mediate the protein degradation process by activating the E3s activity of TTC3, while on the other hand, they may affect protein interactions, eventually leading to a protein homeostasis disorder and the formation of protein aggregates, thereby potentially enhancing the pathogenesis of DS or AD and affecting their common feature, namely cognitive impairment.

### TPR Motif

Proteins contain the TPR motif function in many important activities, such as cell cycle regulation, transcriptional repression, RNA splicing, protein transport, and protein folding. Moreover, the TPR motif is an essential domain that mediates the interaction between proteins (Allan and Ratajczak 2011; Krachler et al. 2010; Zeytuni et al. 2011). TTC3 is a member of the TPR gene family, encoding a matrix protein containing a transmembrane structure; the N-terminal region of the TTC3 protein contains three units of 34 amino acid repeats, similar to the TPR motif (Tsukahara et al. 1998). The N-terminal fragment of TTC3 tends to form aggregates in the nucleus, whereas its C-terminus does not possess this tendency (Gong et al. 2019). The accumulation propensity of the TTC3 N-terminus is likely tied to the TPR motif, although the mechanism of aggregation needs further study.

### RING Finger Domain

The RING finger domain is located in the C-terminus of TTC3 and consists of short motifs rich in cysteine and histidine residues. RING E3s depend on their integrity for biological function (Chaturvedi et al. 2002; Walters et al. 2010) and are the most abundant type of ubiquitin ligases that can mediate the direct transfer of ubiquitin to the substrate; RING E3s can be regulated in different ways, including methylation, phosphorylation, and interaction with small molecules (Morreale and Walden 2016). However, the specific mechanism of how the RING finger domain contributes to the function of TTC3 is still unclear.

### Phosphorylation Site

Phosphorylation and ubiquitination are the two most common posttranslational modifications in eukaryotic proteomes. In most cases, the two modifications occur simultaneously rather than independently, and protein degradation is accomplished by ubiquitination and phosphorylation. Phosphorylation can affect ubiquitination by E3s, and this regulatory process is mainly achieved by phosphorylation of the substrate or the E3s itself (Hunter 2007). TTC3 Ser378 is a possible putative Akt phosphorylation site that is conserved in humans, mice, and rats (Obenauer et al. 2003; Yaffe et al. 2001). Phosphorylation of TTC3 Ser378 is required for TTC3 to perform its function correctly (Suizu et al. 2009; Toker 2009).

### Nuclear Localization Signal (NLS)

The NLS is a protein domain and another form of a signaling peptide and can be regarded as the key for the entry of macromolecular proteins into the nucleus (Bange et al. 2013; Tao et al. 2018). Numerous studies have shown that NLS signaling abnormalities can affect normal physiological functions in many aspects and are associated with a variety of diseases, including neurodegenerative diseases (Nomura et al. 2014). Each type of NLS has similar characteristics but does not have a completely conserved amino acid composition. Different forms of NLSs tend to correspond to different nuclear input mechanisms, and it is possible for the same protein to have several functional types of NLSs simultaneously. Only one functional NLS has been identified for TTC3 (Gong et al. 2019) and is located within amino acid residues 1 to 650 at the N-terminus of TTC3; this NLS guides TTC3 into the nucleus and makes it function as a hub in signal transduction pathways involved in the gene regulation of various pathological processes in DS and AD.

## TTC3 Affects Cognition in DS and AD

Previous studies of patients with AD have revealed similarities in neuropathology between patients from the general population (GP-AD) and those with DS (DS-AD) (Dick et al. 2016). In addition, researchers have found that Ts65Dn mice develop a neuronal endosomal pathology similar to AD that leads to cognitive impairment (Salehi et al. 2006). DS is the most common chromosomal disorder associated with developmental cognitive impairment, whereas *TTC3*, located in the DSCR, can affect neuronal proliferation and differentiation as well as mitochondrial function and may be closely related to cognitive function. We know that DS is mainly caused by the triplication of chromosome 21. In addition to *TTC3*, another star factor, amyloid-β precursor protein (APP), is located on this chromosome. *APP* was one of the first genes identified as a cause of AD (Goate et al. 1991) because it can lead to amyloid accumulation in the brain as early as childhood (Saint-Aubert et al. 2017); therefore, it was considered the main reason for the high incidence of AD in patients with DS in the early years. Some investigators believe that the lack of triplication of *APP* in DS patients does not trigger dementia, emphasizing the critical role of increased *APP* copy number in the development of AD in DS (Head et al. 2018). However, it has been found that patients with phenotypic DS and partial trisomy of chromosome 21 (PT21) lack triploidy of *APP*, suggesting that *APP* copies are not the only mechanism of concurrent dementia in PT21 patients, and in this case, many other genes on chromosome 21 are also included in the trisomy fragment. Moreover, unlike the increased risk of AD found in the majority of DS patients, the risk of AD in these individuals is comparable to that of the general population (Doran et al. 2017; Prasher et al. 1998). In addition, some researchers have proposed that families with three episodes of *APP* alone can develop autosomal dominant early onset AD. Mutations that affect *APP* expression, either regulatory or copy number polymorphisms, affect the pathogenesis of AD patients and families (Rovelet-Lecrux et al. 2006; Sleegers et al. 2006). However, it was later proposed that additional copies of other genes on chromosome 21 increase AD synencephaly pathology and cognitive impairment in mice with DS (Wiseman et al. 2018). Moreover, genome sequencing results of early onset dementia revealed variants of uncertain significance in *TTC3* (Cochran et al. 2019). Therefore, it is likely that *TTC3* is involved in the pathogenesis of DS-AD, but the specific mechanism is unknown. In general, the pathogenesis of DS is an irreversible process; however, the ubiquitination reaction could be reversible, with E3s being among the most critical factors. Both Hs52Sk cells and CMK85 cells are derived from DS individuals (Sato et al. 1989), and *TTC3* expression is upregulated in both abovementioned cell lines, but abnormal expression of TTC3 leads to a phenotype that can be reversed by Myr-Akt in DS cells. TTC3 siRNA can also reverse the DS phenotype because it cannot induce Akt ubiquitination (Suizu et al. 2009). On the other hand, an excess of TTC3 protein induces cytotoxicity, and aggregates formed by TTC3 can cause proteostasis dysregulation, which is consistent with the main pathological changes and characteristics observed in AD (Gong et al. 2019). However, Kohli et al. found that TTC3 may act like APOE in all members of a family with late-onset AD (LOAD), and TTC3 variants may contribute to LOAD risk, but may not initiate AD pathogenesis like APP (Kohli et al. 2016). Thus, it is unclear what role TTC3 plays in it, but it is certain that targeted interventions in the early stages of cognitive impairment can achieve desirable results.

### Affects Neuronal Differentiation via the Rho Pathway

*TTC3* has a mouse ortholog, *mtprd* (Tsukahara et al. 1998), the expression of which is developmentally regulated, and although its expression is ubiquitous at the gross tissue level, the strength of its expression becomes tissue specific during development. The strongest expression is in the nervous system, and appears to be restricted to nonproliferative zones containing differentiated neurons (Lopes et al. 1999). Subsequent, studies in human embryos revealed that, consistent with previous findings, *TTC3* was prevalent in very early embryos but that the strength of the signal gradually increased; at 45 and 50 post-ovulatory (p.o.) days, the strongest signal was found in the nervous system. Furthermore, in the developing nervous system of the fetus, there were regional differences in the intensity of *TTC3* expression, suggesting that *TTC3* expression patterns evolve during development (Rachidi et al. 2000).

The normal morphological development of neurons is essential for the formation of neural networks, and the differentiation of neurons can be divided into processes such as neurogenesis, nerve migration, axon formation, and synaptogenesis, and axonal extension is also associated with a variety of neurological diseases. In the adult mammalian brain, neurogenesis is always present. In the hippocampus, neurogenesis begins with the production of neural progenitor cells (NPCs) in the dentate gyrus, which progressively develop into mature neurons after several rounds of proliferation (Goncalves et al. 2016). The transition of NPCs from a proliferative state to a fully differentiated phenotype is among the most significant events during central nervous system (CNS) development. Evidence suggests that abnormal proliferation of NPCs is one of the causes of cognitive decline in AD (Crews et al. 2010). *TTC3* expression is significantly upregulated in DS both in cells, animal models and in humans, resulting in a range of phenotypes, including cognitive impairment (Guedj et al. 2016; Kong et al. 2014, 2015). However, the increased expression of *TTC3* in DS may be related to the gene’s triploid status, and it has been proposed previously that other triplicated genes in DS may also be involved in the process of neurodegeneration. During development, *TTC3* mRNA is progressively enriched in postmitotic regions of the CNS, thus suggesting its possible involvement in neuronal differentiation (Rachidi et al. 2000).

RhoA is a member of the Rho GTPase family, which is a key regulator of cell dynamics, and the number of axons and dendrites and dendritic arborization during the neuronal growth phase depend on the expression of Rho GTPase (Kim et al. 2017; Maldonado et al. 2017). RhoA signaling has been shown to be involved in a variety of neurodegenerative diseases, including AD (Socodato et al. 2020), and can affect cognitive function (Pearn et al. 2018; Song et al. 2019). Berto et al. suggested that TTC3 inhibits neuronal differentiation through RhoA and Citron kinase (CIT-K) (Berto et al. 2007). RhoA is known to inhibit neurite extension by linking RhoA to neural-specific profilin IIa (PIIa) via RhoA kinase ROCK (da Silva and Dotti 2002; Stiess and Bradke 2011). At the same time, TPR motif-containing proteins can physically interact with themselves, with each other, and with the cytoskeleton through their TPR motifs (Berto et al. 2014; Eki et al. 1997). Therefore, TTC3 overexpression may lead to an imbalance of protein–protein interactions during cell growth and differentiation. In fact, inhibition of RhoA and ROCK and knockout of Citron N (CIT-N) can rescue the neuronal growth phenotype induced by TTC3 overexpression. In this case, however, ROCK regulates neurite extension downstream of CIT-N rather than parallel to it.

It is noteworthy that activated RhoA is specifically associated with the Golgi apparatus of differentiated neurons (Camera et al. 2003), and it may regulate Golgi compactness through CIT-N, a central nervous system-specific variant of the cytokinesis regulator CIT-K (Furuyashiki et al. 1999; Madaule et al. 1998). TTC3 overexpression may affect Golgi organization through two main pathways: a "canonical" pathway from RhoA to actin polymerization through ROCK and PIIa, and a noncanonical pathway involving CIT-N and RhoA. Citron was recently found to associate with microtubules (Bassi et al. 2013), and there is a decisive role for microtubules in the Golgi. Increased actin polymerization is also involved in the Golgi fragmentation phenotype produced by TTC3 overexpression. However, the inhibitory effect of TTC3 overexpression on neurite extension can be reversed by CIT-K RNAi (Berto et al. 2007). Knockdown of PIIa can also rescue the neurite elongation and Golgi phenotypes induced by TTC3 overexpression (Berto et al. 2014). The above reports suggest that normal growth and development of neurons may be regulated by targeting TTC3 to avoid impaired cognitive function.

### Affects Neuronal Proliferation via Akt Signaling

AKT, also known as protein kinase B (PKB), and activated Akt regulate the function of cells by phosphorylating downstream factors such as a variety of enzymes, kinases, and transcription factors. In the absence of AKT signaling inhibition, the cell cycle interval of neural progenitor cells is accelerated and rapidly enters the division phase, which accelerates the proliferation and differentiation of neuronal cells and plays an important role in the regulation of neurogenesis and neural cell proliferation and differentiation(Polchi et al. 2018; Zhai et al. 2019). Solzak et al. (Solzak et al. 2013) showed that trisomic TTC3 is overexpressed in DS embryos and may affect nuclear phospho-Akt localization and cell survival. As described earlier, TTC3 is an E3s, while Akt is its specific substrate. TTC3 can utilize its E3s activity to ubiquitinate Akt, thereby affecting the PI3-kinase (PI3K)/Akt pathway and stimulating Akt degradation (Suizu et al. 2009). When TTC3 expression is increased in DS cells, phosphorylated Akt levels are decreased, resulting in relative accumulation of cells in the G2 M phase of the cell cycle. In other words, overexpression of TTC3 can promote apoptosis, while inhibition of TTC3 can enhance cell proliferation. While Akt is at the intersection of several signaling pathways (Hers et al. 2011; Zhang et al. 2011), TTC3 regulates transcription factors such as cAMP response element-binding protein (CREB) through phospho-Akt, which affects cell survival, proliferation, and differentiation (Carloni et al. 2010; Solzak et al. 2013). Impaired proliferation and neuronal differentiation ability may affect the maturation of the hippocampus, which leads to cognitive decline, suggesting that regulating TTC3 may be a potential strategy for neuroprotection. In addition, tau, one of the culprits of AD, is also one of the phosphorylated substrates of AKT, and AKT hyperactivation of AD, can promote tau hyperphosphorylation (Cao et al. 2020). Thus, whether there is a correlation between TTC3 and tau remains to be determined.

### Affects Mitochondrial Function in Neurons via POLG Ubiquitination

DNA polymerase γ (POLG), the only enzyme known to be located in mitochondria involved in mitochondrial DNA replication and repair, is critical to maintain cellular function and integrity. POLG has three enzymatic activities, namely, DNA polymerase activity, exonuclease activity and lyase activity; therefore, when POLG is mutated, mitochondrial function is damaged, and energy-consuming organs such as the brain are affected (Kujoth et al. 2005; Rahman and Copeland 2019). Some evidence has shown that the overexpression of TTC3 can prevent the extension of neurites through the regulation of POLG (Gong et al. 2017). When TTC3 is slightly overexpressed, soluble functional TTC3 promotes POLG degradation, leading to a decrease in functional POLG. When TTC3 is overexpressed at high levels, it will form aggregates, interrupt the degradation of POLG, and isolate POLG into aggregates, resulting in the loss of its function. POLG is also included in TTC3 aggregates caused by high TTC3 protein expression.

**Fig. 3:**
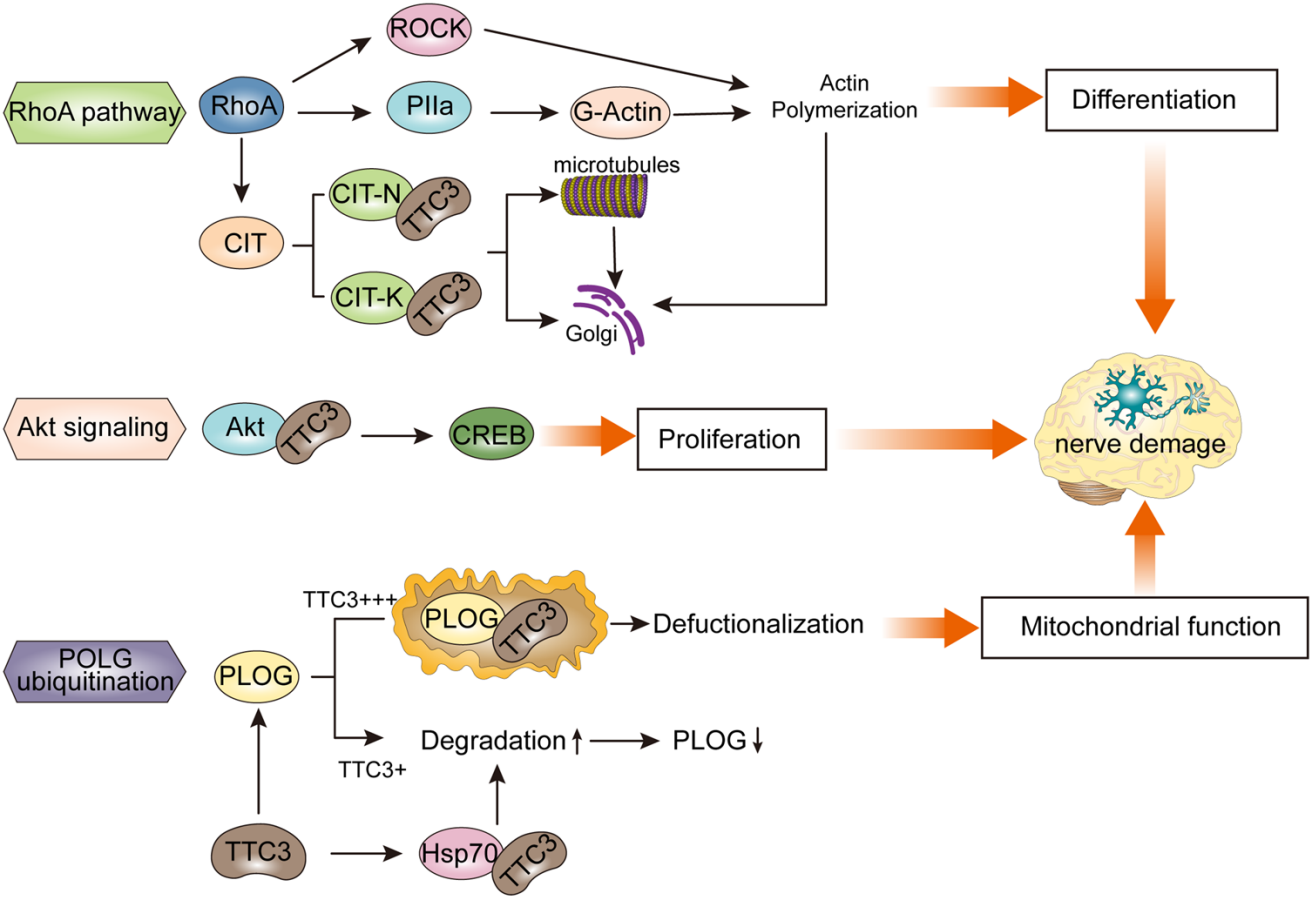
Role of TTC3 in cognitive impairment TTC3 is involved in the proliferation and differentiation of nerve cells through the regulation of the RhoA, ROCK, CIT-N, PIIa, and Akt signaling pathways and affects mitochondrial function by POLG ubiquitination, which may eventually lead to cognitive impairment

In addition, TTC3 could alleviate aggregation and promote the degradation of POLG with coexpression with Hsp70. Heat shock proteins (HSPs) are chaperones involved in proteasomal degradation. It has been reported that Hsp70 can affect the degradation of tau by interacting with tau, which leads to cognitive decline (Choi et al. 2020). From this point of view, TTC3 may affect mitochondrial function by affecting POLG ubiquitination, ultimately affecting cognitive function (Fig. 3).

## PQC Mechanisms of TTC3-Linked Cognitive Impairment

**Fig. 4:**
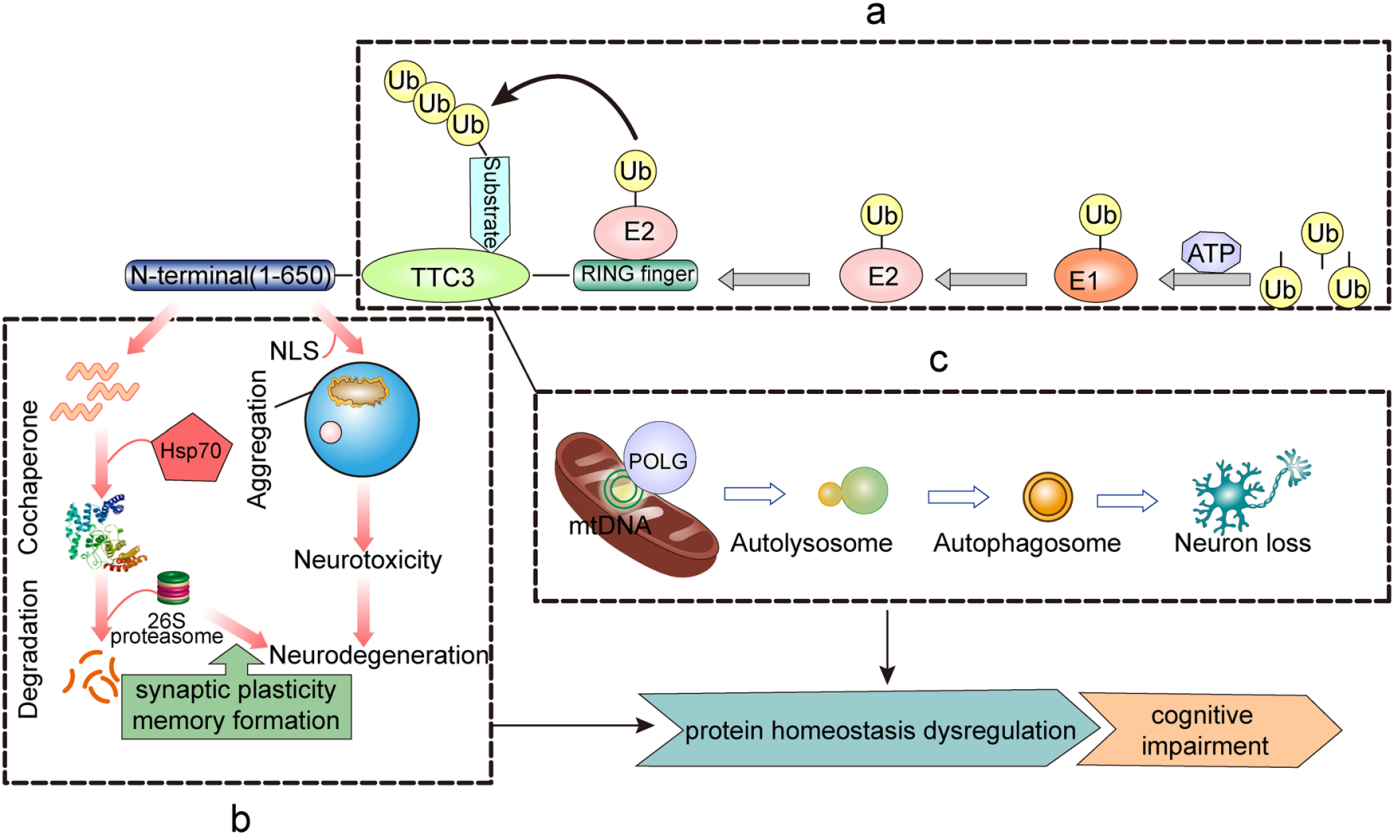
TTC3-mediated PQC mechanism **a** TTC3 acts as a cochaperone involved in the ubiquitin-protease degradation process and enters the nucleus to form aggregates mediated by the NLS, with the N-terminus playing a key role. **b** TTC3 is involved in the ubiquitination process as an E3 ubiquitin ligase, and the RING finger domain is the critical element of TTC3 function. **c** TTC3 affects mitochondrial function by regulating POLG, which promotes autophagy and ultimately neuronal loss

TTC3 is closely related to proteostasis, and it has been suggested that TTC3 may be one of the risk factors for cognition (Lott and Head 2019). Ubiquitination and proteasome-mediated degradation play important roles in the regulation of protein homeostasis. It has been found in animal models that early impairment of the UPS and loss of cellular proteostasis may be major mediators of neurodegeneration and cognitive impairment, whereas impaired substrate ubiquitination and proteasomal degradation may play roles in proteostasis dysregulation. Corpas et al. proposed that enhanced proteostasis could increase the brain’s resistance to neurodegeneration (Corpas et al. 2019). Protein homeostasis is disrupted in both DS and AD (Chen et al. 2020; Tramutola et al. 2018), allowing for the accumulation of intracellular deposits of misfolded proteins and protein-toxic peptides. However, PQC can use various E3 ubiquitin ligases to selectively degrade abnormal proteins and counteract the hazards caused by protein misfolding (Kanack et al. 2018). As an adaptor molecule between ubiquitin and protein substrates, E3s can specifically recognize substrates and dominate the ubiquitination modification of proteins, thereby affecting protein homeostasis. It has been deduced from the phenotypes that develop that TTC3 can affect the protein degradation process and form aggregates, facilitate the treatment of misfolded proteins as molecular chaperone cofactors, and impact mitochondrial function (Fig. 4).

### Misfolded Protein and Cochaperone

Approximately one-third of newly synthesized proteins in human cells must be removed due to incorrect folding, and misfolded proteins form aggregates if they are not removed in a timely and effective manner (Gandhi et al. 2019). Cochaperone mainly assist in the correct folding of proteins, and in addition to having a domain that can bind to chaperones, they have a domain associated with ubiquitin–proteasome degradation, which is either a fragment that can interact directly with the proteasome or a domain with ubiquitin ligase function. Heat shock proteins (HSPs), such as Hsp70 and Hsp90, are one of the most important molecular chaperones. In most tissues, the expression of HSPs decreases with age, which leads to misfolded protein accumulation. Thus, as a protein with the dual identity of an E3s and a molecular cochaperone, TTC3 connects the molecular chaperone and the UPS, which may be involved in maintaining protein homeostasis by binding to Hsp70 (McClellan et al. 2005). In addition, it has been reported that the two TPR motif-containing cochaperone in Hsp90 can interact simultaneously (Li et al. 2012) and that tau protein can be regulated by competing for binding to Hsp90 (Hildenbrand et al. 2011; Shelton et al. 2017). According to the above mechanism, it is unclear whether TTC3, which also has a TPR motif, can also affect the expression level of tau by binding to Hsp90.

### Protein Degradation and Aggregates in UPS

The UPS is a member of the two major systems for maintaining protein homeostasis in cells, and it is responsible for 80–85% of protein degradation in eukaryotes via the ubiquitin–proteasome pathway (UPP). Abnormalities in any part of the UPP may translate into the accumulation of abnormal proteins in cells, and neurodegeneration is essentially caused by protein accumulation due to protein misfolding. A correlation between tau pathology and UPS dysfunction has been demonstrated (Ciechanover and Kwon 2015), with either phosphorylated or no phosphorylated tau being degraded by the 26S proteasome (Cripps et al. 2006). Moreover, ubiquitin accumulation is observed in the AD brain (Mori et al. 1987). In addition, a role for the UPS in DS has been demonstrated, and reduced levels of protein ubiquitination have been observed in human DS cell lines (Granese et al. 2013), whereas inhibition of the UPS has been found to induce neuronal degeneration and death in a mouse model of DS (Necchi et al. 2011). In other research, it has been suggested that increased ubiquitin–proteasome activity in the brain is closely associated with both synaptic plasticity and memory formation (McFadden et al. 2019).

Under normal circumstances, the TTC3 protein exists in a nearly full-length form to assist in maintaining protein homeostasis. However, when protein homeostasis is disrupted, abnormal cleavage and aggregation of the TTC3 protein may occur. In neurodegenerative diseases, activation of certain proteolytic enzymes may also lead to aberrant cleavage of TTC3. Then, N-terminal proteolytic fragments enter the nucleus and form aggregates with the help of the NLS (Gong et al. 2019). When TTC3 is overexpressed to form aggregates, physiological function is lost, and TTC3 aggregation induces neurotoxicity and further deteriorates proteostasis, thereby forming a vicious cycle.

### Mitochondrial Dysfunction and Autophagy

Damage to mitochondrial function not only affects mitochondrial functional processes but also leads to the blockage of protein entry into the mitochondria, thereby allowing mitochondrial precursor proteins to accumulate in the cytoplasm, leading to protein imbalance. The POLG protein is the catalytic subunit of DNA polymerase, the only polymerase found in the mitochondria of animal cells and involved in the maintenance of various mitochondrial functions. It is possible that TTC3 affects mitochondrial function by regulating POLG, and severe mitochondrial damage then initiates the mitophagy process. POLG can affect the initial amplification of mtDNA, resulting in neuronal loss (Tzoulis et al. 2014). The loss of interneurons and severe respiratory defects of the remaining interneurons together lead to impaired neural network oscillations, which may lead to neurological deficits, such as cognitive impairment(Lax et al. 2016).

## Conclusion and Perspective

TTC3 is involved in the pathogenesis of cognitive impairment owing to its functional structure and roles in PQC. Notably, the proteostasis manipulated by PQC activity is involved from the beginning of disease progression, and the specific mechanism remains to be further explored. These mechanisms are not necessarily parallel, as each has its own specific responsibilities, but they are likely to intersect each other. However, studies examining homozygous cells have not been performed because *TTC3* homozygous knockout (*TTC3* − / −) is lethal, thus partially limiting the progression of studies on TTC3. In the limited literature available, it has been suggested that TTC3 is linked to ubiquitin on the ring finger domain and then activates E3S activity, while TPR motifs can interact with proteins to affect disease pathogenesis, the role of TTC3 in cognition has not been elucidated. Furthermore, specific information regarding the TTC3 phosphorylation site and NLS is unknown and needs to be studied in depth. The overall prognosis of DS-AD remains poor, which seriously affects the quality of life of patients and increases the socioeconomic burden, and the prevalence of these diseases is showing an upward trend. However, the expression of TTC3 and the induced phenotype can be halted. Further research progress on TTC3 in nerve growth and cognitive impairment could provide a better understanding of the role of TTC3 in neurobiology and as a potential target for the treatment of such diseases.

## References

[CR1] Allan RK, Ratajczak T (2011). Versatile TPR domains accommodate different modes of target protein recognition and function. Cell Stress Chaperones.

[CR2] Arron JR (2006). NFAT dysregulation by increased dosage of DSCR1 and DYRK1A on chromosome 21. Nature.

[CR3] Bange G, Murat G, Sinning I, Hurt E, Kressler D (2013). New twist to nuclear import: When two travel together. Commun Integr Biol.

[CR4] Bassi ZI, Audusseau M, Riparbelli MG, Callaini G, D'Avino PP (2013). Citron kinase controls a molecular network required for midbody formation in cytokinesis. Proc Natl Acad Sci U S A.

[CR5] Berto G (2007). The Down syndrome critical region protein TTC3 inhibits neuronal differentiation via RhoA and Citron kinase. J Cell Sci.

[CR6] Berto GE (2014). The DCR protein TTC3 affects differentiation and Golgi compactness in neurons through specific actin-regulating pathways. PLoS ONE.

[CR7] Camera P (2003). Citron-N is a neuronal Rho-associated protein involved in Golgi organization through actin cytoskeleton regulation. Nat Cell Biol.

[CR8] Cao Y (2020). Inhibition of mTORC1 improves STZ-induced AD-like impairments in mice. Brain Res Bull.

[CR9] Carloni S, Girelli S, Scopa C, Buonocore G, Longini M, Balduini W (2010). Activation of autophagy and Akt/CREB signaling play an equivalent role in the neuroprotective effect of rapamycin in neonatal hypoxia-ischemia. Autophagy.

[CR10] Chaturvedi P (2002). Chfr regulates a mitotic stress pathway through its RING-finger domain with ubiquitin ligase activity. Cancer Res.

[CR11] Chen XQ (2020). Targeting increased levels of APP in Down syndrome: Posiphen-mediated reductions in APP and its products reverse endosomal phenotypes in the Ts65Dn mouse model. Alzheimers Dement.

[CR12] Choi H (2020). Acetylation changes tau interactome to degrade tau in Alzheimer's disease animal and organoid models. Aging Cell.

[CR13] Ciechanover A, Kwon YT (2015). Degradation of misfolded proteins in neurodegenerative diseases: therapeutic targets and strategies. Exp Mol Med.

[CR14] Ciechanover A, Kwon YT (2017). Protein Quality Control by Molecular Chaperones in Neurodegeneration. Front Neurosci.

[CR15] Cochran JN (2019). Genome sequencing for early-onset or atypical dementia: high diagnostic yield and frequent observation of multiple contributory alleles Cold Spring Harb. Mol Case Stud.

[CR16] Corpas R, Grinan-Ferre C, Rodriguez-Farre E, Pallas M, Sanfeliu C (2019). Resveratrol Induces Brain Resilience Against Alzheimer Neurodegeneration Through Proteostasis Enhancement. Mol Neurobiol.

[CR17] Costa-Mattioli M, Walter P (2020). The integrated stress response: From mechanism to disease. Science.

[CR18] Crews L (2010). Increased BMP6 levels in the brains of Alzheimer's disease patients and APP transgenic mice are accompanied by impaired neurogenesis. J Neurosci.

[CR19] Cripps D, Thomas SN, Jeng Y, Yang F, Davies P, Yang AJ (2006). Alzheimer disease-specific conformation of hyperphosphorylated paired helical filament-Tau is polyubiquitinated through Lys-48, Lys-11, and Lys-6 ubiquitin conjugation. J Biol Chem.

[CR20] Cristofani R (2017). Inhibition of retrograde transport modulates misfolded protein accumulation and clearance in motoneuron diseases. Autophagy.

[CR21] da Silva JS, Dotti CG (2002). Breaking the neuronal sphere: regulation of the actin cytoskeleton in neuritogenesis. Nat Rev Neurosci.

[CR22] Dahmane N (1998). Transcriptional map of the 2.5-Mb CBR-ERG region of chromosome 21 involved in Down syndrome. Genomics.

[CR23] Delabar JM (1993). Molecular mapping of twenty-four features of Down syndrome on chromosome 21. Eur J Hum Genet.

[CR24] Dick MB, Doran E, Phelan M, Lott IT (2016). Cognitive Profiles on the Severe Impairment Battery Are Similar in Alzheimer Disease and Down Syndrome With Dementia. Alzheimer Dis Assoc Disord.

[CR25] Dikic I (2017). Proteasomal and Autophagic Degradation Systems. Annu Rev Biochem.

[CR26] Doran E (2017). Down Syndrome, Partial Trisomy 21, and Absence of Alzheimer's Disease: The Role of APP. J Alzheimers Dis.

[CR27] Eki T (1997). Cloning and characterization of novel gene, DCRR1, expressed from Down's syndrome critical region of human chromosome 21q22.2. DNA Seq.

[CR28] Enam C, Geffen Y, Ravid T, Gardner RG (2018). Protein Quality Control Degradation in the Nucleus. Annu Rev Biochem.

[CR29] Freilich R, Arhar T, Abrams JL, Gestwicki JE (2018). Protein-Protein Interactions in the Molecular Chaperone Network. Acc Chem Res.

[CR30] Furuyashiki T (1999). Citron, a Rho-target, interacts with PSD-95/SAP-90 at glutamatergic synapses in the thalamus. J Neurosci.

[CR31] Gandhi J (2019). Protein misfolding and aggregation in neurodegenerative diseases: a review of pathogeneses, novel detection strategies, and potential therapeutics. Rev Neurosci.

[CR32] Goate A (1991). Segregation of a missense mutation in the amyloid precursor protein gene with familial Alzheimer's disease. Nature.

[CR33] Goncalves JT, Schafer ST, Gage FH (2016). Adult Neurogenesis in the Hippocampus: From Stem Cells to Behavior. Cell.

[CR34] Gong Y, Wang X, Shang X, Xiao SP, Li W, Shang Y, Dou F (2017). Tetratricopeptide repeat domain 3 overexpression tends to form aggregates and inhibit ubiquitination and degradation of DNA polymerase gamma. Oncotarget.

[CR35] Gong Y, Wang K, Xiao SP, Mi P, Li W, Shang Y, Dou F (2019). Overexpressed TTC3 Protein Tends to be Cleaved into Fragments and Form Aggregates in the Nucleus. Neuromolecular Med.

[CR36] Granese B (2013). Validation of microarray data in human lymphoblasts shows a role of the ubiquitin-proteasome system and NF-kB in the pathogenesis of Down syndrome. BMC Med Genomics.

[CR37] Guedj F, Pennings JL, Massingham LJ, Wick HC, Siegel AE, Tantravahi U, Bianchi DW (2016). An Integrated Human/Murine Transcriptome and Pathway Approach To Identify Prenatal Treatments For Down Syndrome. Sci Rep.

[CR38] Harris LD, Jasem S, Licchesi JDF (2020). The Ubiquitin System in Alzheimer's Disease. Adv Exp Med Biol.

[CR39] Hattori M (2000). The DNA sequence of human chromosome 21. Nature.

[CR40] Head E, Helman AM, Powell D, Schmitt FA (2018). Down syndrome, beta-amyloid and neuroimaging. Free Radic Biol Med.

[CR41] Hers I, Vincent EE, Tavare JM (2011). Akt signalling in health and disease. Cell Signal.

[CR42] Hildenbrand ZL, Molugu SK, Herrera N, Ramirez C, Xiao C, Bernal RA (2011). Hsp90 can accommodate the simultaneous binding of the FKBP52 and HOP proteins. Oncotarget.

[CR43] Hipp MS, Kasturi P, Hartl FU (2019). The proteostasis network and its decline in ageing. Nat Rev Mol Cell Biol.

[CR44] Hunter T (2007). The age of crosstalk: phosphorylation, ubiquitination, and beyond. Mol Cell.

[CR45] Imai J, Yashiroda H, Maruya M, Yahara I, Tanaka K (2003). Proteasomes and molecular chaperones: cellular machinery responsible for folding and destruction of unfolded proteins. Cell Cycle.

[CR46] Joshi V, Amanullah A, Upadhyay A, Mishra R, Kumar A, Mishra A (2016). A Decade of Boon or Burden: What Has the CHIP Ever Done for Cellular Protein Quality Control Mechanism Implicated in Neurodegeneration and Aging?. Front Mol Neurosci.

[CR47] Kabir MT (2020). Evidence Linking Protein Misfolding to Quality Control in Progressive Neurodegenerative Diseases. Curr Top Med Chem.

[CR48] Kanack AJ, Newsom OJ, Scaglione KM (2018). Most mutations that cause spinocerebellar ataxia autosomal recessive type 16 (SCAR16) destabilize the protein quality-control E3 ligase CHIP. J Biol Chem.

[CR49] Kim HJ (2017). Regulation of RhoA activity by the cellular prion protein. Cell Death Dis.

[CR50] Kim JH, Ham S, Lee Y, Suh GY, Lee YS (2019). TTC3 contributes to TGF-beta1-induced epithelial-mesenchymal transition and myofibroblast differentiation, potentially through SMURF2 ubiquitylation and degradation. Cell Death Dis.

[CR51] Kohli MA (2016). Segregation of a rare TTC3 variant in an extended family with late-onset Alzheimer disease. Neurol Genet.

[CR52] Kong XD, Liu N, Xu XJ (2014). Bioinformatics analysis of biomarkers and transcriptional factor motifs in Down syndrome. Braz J Med Biol Res.

[CR53] Kong XD, Liu N, Xu XJ, Zhao ZH, Jiang M (2015). Screening of human chromosome 21 genes in the dorsolateral prefrontal cortex of individuals with Down syndrome. Mol Med Rep.

[CR54] Kong D (2020). Curcumin blunts epithelial-mesenchymal transition of hepatocytes to alleviate hepatic fibrosis through regulating oxidative stress and autophagy. Redox Biol.

[CR55] Krachler AM, Sharma A, Kleanthous C (2010). Self-association of TPR domains: Lessons learned from a designed, consensus-based TPR oligomer. Proteins.

[CR56] Krstic D, Knuesel I (2013). Deciphering the mechanism underlying late-onset Alzheimer disease. Nat Rev Neurol.

[CR57] Kujoth GC (2005). Mitochondrial DNA mutations, oxidative stress, and apoptosis in mammalian aging. Science.

[CR58] Lamb JR, Tugendreich S, Hieter P (1995). Tetratrico peptide repeat interactions: to TPR or not to TPR?. Trends Biochem Sci.

[CR59] Lax NZ (2016). Extensive respiratory chain defects in inhibitory interneurones in patients with mitochondrial disease. Neuropathol Appl Neurobiol.

[CR60] Li J, Soroka J, Buchner J (2012). The Hsp90 chaperone machinery: conformational dynamics and regulation by co-chaperones. Biochim Biophys Acta.

[CR61] Lopes C, Rachidi M, Gassanova S, Sinet PM, Delabar JM (1999). Developmentally regulated expression of mtprd, the murine ortholog of tprd, a gene from the Down syndrome chromosomal region 1. Mech Dev.

[CR62] Lott IT, Head E (2019). Dementia in Down syndrome: unique insights for Alzheimer disease research. Nat Rev Neurol.

[CR63] Lu H (2017). Sevoflurane Acts on Ubiquitination-Proteasome Pathway to Reduce Postsynaptic Density 95 Protein Levels in Young Mice. Anesthesiology.

[CR64] Lyle R (2009). Genotype-phenotype correlations in Down syndrome identified by array CGH in 30 cases of partial trisomy and partial monosomy chromosome 21. Eur J Hum Genet.

[CR65] Madaule P (1998). Role of citron kinase as a target of the small GTPase Rho in cytokinesis. Nature.

[CR66] Maldonado H (2017). Astrocyte-to-neuron communication through integrin-engaged Thy-1/CBP/Csk/Src complex triggers neurite retraction via the RhoA/ROCK pathway. Biochim Biophys Acta Mol Cell Res.

[CR67] Mann DM (1988). The pathological association between Down syndrome and Alzheimer disease. Mech Ageing Dev.

[CR68] Martinez-Cue C, Rueda N (2020). Cellular Senescence in Neurodegenerative Diseases. Front Cell Neurosci.

[CR69] McClellan AJ, Tam S, Kaganovich D, Frydman J (2005). Protein quality control: chaperones culling corrupt conformations. Nat Cell Biol.

[CR70] McFadden T, Devulapalli RK, Jarome TJ (2019). Quantifying Subcellular Ubiquitin-proteasome Activity in the Rodent Brain. J Vis Exp.

[CR71] Mishra R, Upadhyay A, Prajapati VK, Dhiman R, Poluri KM, Jana NR, Mishra A (2019). LRSAM1 E3 ubiquitin ligase: molecular neurobiological perspectives linked with brain diseases. Cell Mol Life Sci.

[CR72] Montoya JC, Fajardo D, Pena A, Sanchez A, Dominguez MC, Satizabal JM, Garcia-Vallejo F (2014). Global differential expression of genes located in the Down Syndrome Critical Region in normal human brain. Colomb Med (Cali).

[CR73] Mori H, Kondo J, Ihara Y (1987). Ubiquitin is a component of paired helical filaments in Alzheimer's disease. Science.

[CR74] Morreale FE, Walden H (2016). Types of Ubiquitin Ligases Cell.

[CR75] Necchi D, Lomoio S, Scherini E (2011). Dysfunction of the ubiquitin-proteasome system in the cerebellum of aging Ts65Dn mice. Exp Neurol.

[CR76] Nomura T, Watanabe S, Kaneko K, Yamanaka K, Nukina N, Furukawa Y (2014). Intranuclear aggregation of mutant FUS/TLS as a molecular pathomechanism of amyotrophic lateral sclerosis. J Biol Chem.

[CR77] Obenauer JC, Cantley LC, Yaffe MB (2003). Scansite 2.0: Proteome-wide prediction of cell signaling interactions using short sequence motifs. Nucleic Acids Res.

[CR78] Ohira M (1996). Identification of a novel human gene containing the tetratricopeptide repeat domain from the Down syndrome region of chromosome 21. DNA Res.

[CR79] Park J, Chung KC (2013). New Perspectives of Dyrk1A Role in Neurogenesis and Neuropathologic Features of Down Syndrome. Exp Neurobiol.

[CR80] Pearn ML (2018). Inhibition of RhoA reduces propofol-mediated growth cone collapse, axonal transport impairment, loss of synaptic connectivity, and behavioural deficits. Br J Anaesth.

[CR81] Polchi A, Magini A, Meo DD, Tancini B, Emiliani C (2018). mTOR Signaling and Neural Stem Cells: The Tuberous Sclerosis Complex Model. Int J Mol Sci.

[CR82] Prasher VP, Farrer MJ, Kessling AM, Fisher EM, West RJ, Barber PC, Butler AC (1998). Molecular mapping of Alzheimer-type dementia in Down's syndrome. Ann Neurol.

[CR83] Rachidi M (2000). Regional and cellular specificity of the expression of TPRD, the tetratricopeptide Down syndrome gene, during human embryonic development. Mech Dev.

[CR84] Rahman S, Copeland WC (2019). POLG-related disorders and their neurological manifestations. Nat Rev Neurol.

[CR85] Ronan A, Fagan K, Christie L, Conroy J, Nowak NJ, Turner G (2007). Familial 4.3 Mb duplication of 21q22 sheds new light on the Down syndrome critical region. J Med Genet.

[CR86] Rovelet-Lecrux A (2006). APP locus duplication causes autosomal dominant early-onset Alzheimer disease with cerebral amyloid angiopathy. Nat Genet.

[CR87] Saint-Aubert L, Lemoine L, Chiotis K, Leuzy A, Rodriguez-Vieitez E, Nordberg A (2017). Tau PET imaging: present and future directions. Mol Neurodegener.

[CR88] Salehi A (2006). Increased App expression in a mouse model of Down's syndrome disrupts NGF transport and causes cholinergic neuron degeneration. Neuron.

[CR89] Samant RS, Livingston CM, Sontag EM, Frydman J (2018). Distinct proteostasis circuits cooperate in nuclear and cytoplasmic protein quality control. Nature.

[CR90] Sato T (1989). Establishment of a human leukaemic cell line (CMK) with megakaryocytic characteristics from a Down's syndrome patient with acute megakaryoblastic leukaemia. Br J Haematol.

[CR91] Shelton LB, Koren J, Blair LJ (2017). Imbalances in the Hsp90 Chaperone Machinery: Implications for Tauopathies. Front Neurosci.

[CR92] Sinet PM (1994). Mapping of the Down syndrome phenotype on chromosome 21 at the molecular level. Biomed Pharmacother.

[CR93] Sleegers K (2006). APP duplication is sufficient to cause early onset Alzheimer's dementia with cerebral amyloid angiopathy. Brain.

[CR94] Smith DJ (1997). Functional screening of 2 Mb of human chromosome 21q22.2 in transgenic mice implicates minibrain in learning defects associated with Down syndrome. Nat Genet.

[CR95] Socodato R (2020). Microglia Dysfunction Caused by the Loss of Rhoa Disrupts Neuronal Physiology and Leads to Neurodegeneration. Cell Rep.

[CR96] Solzak JP, Liang Y, Zhou FC, Roper RJ (2013). Commonality in Down and fetal alcohol syndromes. Birth Defects Res A Clin Mol Teratol.

[CR97] Song X (2019). Protective effects of the ROCK inhibitor fasudil against cognitive dysfunction following status epilepticus in male rats. J Neurosci Res.

[CR98] Startin CM (2019). Cognitive markers of preclinical and prodromal Alzheimer's disease in Down syndrome. Alzheimers Dement.

[CR99] Stiess M, Bradke F (2011). Neuronal polarization: the cytoskeleton leads the way. Dev Neurobiol.

[CR100] Suizu F (2009). The E3 ligase TTC3 facilitates ubiquitination and degradation of phosphorylated Akt. Dev Cell.

[CR101] Sun L (2014). Rapid detection of Down's syndrome using quantitative real-time PCR (qPCR) targeting segmental duplications on chromosomes 21 and 11. Gene.

[CR102] Tao H, Zhou X, Zhao B, Li KS (2018). Conflicting Effects of Methylglyoxal and Potential Significance of miRNAs for Seizure Treatment. Front Mol Neurosci.

[CR103] Tiernan CT (2016). Protein homeostasis gene dysregulation in pretangle-bearing nucleus basalis neurons during the progression of Alzheimer's disease. Neurobiol Aging.

[CR104] Toker A (2009). TTC3 ubiquitination terminates Akt-ivation. Dev Cell.

[CR105] Tramutola A (2018). Intranasal rapamycin ameliorates Alzheimer-like cognitive decline in a mouse model of Down syndrome. Transl Neurodegener.

[CR106] Tsukahara F, Hattori M, Muraki T, Sakaki Y (1996). Identification and cloning of a novel cDNA belonging to tetratricopeptide repeat gene family from Down syndrome-critical region 21q22.2. J Biochem.

[CR107] Tsukahara F (1998). Molecular characterization of the mouse mtprd gene, a homologue of human TPRD: unique gene expression suggesting its critical role in the pathophysiology of Down syndrome. J Biochem.

[CR108] Tzoulis C (2014). Molecular pathogenesis of polymerase gamma-related neurodegeneration. Ann Neurol.

[CR109] Uddin MS (2020). Emerging Proof of Protein Misfolding and Interaction in Multifactorial Alzheimer's Disease. Curr Top Med Chem.

[CR110] Verheijen BM, Oyanagi K, van Leeuwen FW (2018). Dysfunction of Protein Quality Control in Parkinsonism-Dementia Complex of Guam. Front Neurol.

[CR111] Walters MS, Kyratsous CA, Silverstein SJ (2010). The RING finger domain of Varicella-Zoster virus ORF61p has E3 ubiquitin ligase activity that is essential for efficient autoubiquitination and dispersion of Sp100-containing nuclear bodies. J Virol.

[CR112] Wei J, Xiong Z, Lee JB, Cheng J, Duffney LJ, Matas E, Yan Z (2016). Histone Modification of Nedd4 Ubiquitin Ligase Controls the Loss of AMPA Receptors and Cognitive Impairment Induced by Repeated Stress. J Neurosci.

[CR113] Wiseman FK (2015). A genetic cause of Alzheimer disease: mechanistic insights from Down syndrome. Nat Rev Neurosci.

[CR114] Wiseman FK (2018). Trisomy of human chromosome 21 enhances amyloid-beta deposition independently of an extra copy of APP. Brain.

[CR115] Yaffe MB, Leparc GG, Lai J, Obata T, Volinia S, Cantley LC (2001). A motif-based profile scanning approach for genome-wide prediction of signaling pathways. Nat Biotechnol.

[CR116] Zeytuni N (2011). Self-recognition mechanism of MamA, a magnetosome-associated TPR-containing protein, promotes complex assembly. Proc Natl Acad Sci U S A.

[CR117] Zhai Y, Ma Y, Liu J, Zhu Y, Xie K, Yu L, Zhang H (2019). Brain-Derived Neurotrophic Factor Alleviates Ropivacaine-Induced Neuronal Damage by Enhancing the Akt Signaling Pathway. Med Sci Monit.

[CR118] Zhang X, Tang N, Hadden TJ, Rishi AK (2011). Akt, FoxO and regulation of apoptosis. Biochim Biophys Acta.

[CR119] Zis P, Strydom A (2018). Clinical aspects and biomarkers of Alzheimer's disease in Down syndrome. Free Radic Biol Med.

